# Postmortem Detection of “Clinically Undiagnosed” Diffuse Large B-Cell Lymphoma: Gross and Microscopic Findings

**DOI:** 10.3390/diagnostics14171901

**Published:** 2024-08-29

**Authors:** Vincenzo Cianci, Daniela Sapienza, Giovanni Bartoloni, Alessio Cianci, Annalisa Cracò, Fausto Omero, Patrizia Gualniera, Alessio Asmundo, Cristina Mondello

**Affiliations:** 1 Department of Biomedical and Dental Sciences and Morphofunctional Imaging, Section of Legal Medicine, University of Messina, via Consolare Valeria 1, 98125 Messina, Italy; daniela.sapienza@unime.it (D.S.); patrizia.gualniera@unime.it (P.G.); alessio.asmundo@unime.it (A.A.); 2 Department of Anatomy, Diagnostic Pathology, Legal Medicine Hygiene and Public Health, University of Catania, 95122 Catania, Italy; gbartoloni@unict.it; 3 Department of Cardiovascular Medicine, Fondazione Policlinico Universitario A. Gemelli-IRCCS, Largo A. Gemelli 8, 00168 Rome, Italy; alessiocianci.1998@gmail.com; 4 Department of Biomedical Sciences and Morphological and Functional Imaging, Diagnostic and Interventional Radiology Unit, University Hospital Messina, 98125 Messina, Italy; annalisacraco@hotmail.it; 5 Medical Oncology Unit, Department of Human Pathology “G. Barresi”, University of Messina, 98125 Messina, Italy; faustoomero@hotmail.it

**Keywords:** forensic pathology, forensic autopsy, large B-cell lymphoma, AIDS, postmortem diagnosis

## Abstract

Diffuse large B-cell lymphoma is considered the most found non-Hodgkin lymphoma in adults. Diffuse large B-cell lymphoma, which also occurs in sporadic forms, is associated with some pathological conditions, including human immunodeficiency virus infection, especially if it progresses to AIDS. The authors report the case of a 45-year-old man with AIDS in whom a postmortem diagnosis of diffuse large B-cell lymphoma was performed. The proposed images document extensive pluri-visceral involvement, already visible macroscopically, and subsequently confirmed through histological examination.

The authors report a rare case of postmortem diagnosis of diffuse large B-cell lymphoma (DLBCL), undiagnosed during life, characterized by an evident pluri-visceral involvement. The case concerns a 45-year-old man found dead at home. On request of the Judicial Authority, an autopsy was performed. Anamnestically, it appeared that the subject was suffering from AIDS (acquired immunodeficiency syndrome) with a CD4/CD8 ratio of 0.18, obesity, dyslipidemia, diabetes mellitus, arterial hypertension, chronic ischemic heart disease, chronic obstructive pulmonary disease with chronic respiratory failure under oxygen therapy treatment, chronic renal failure and neurological disorders, mainly represented by personality disorder and epilepsy.

The human immunodeficiency virus (HIV) is a retrovirus, capable of recognizing and binding the CD4 receptor on the surface of T-lymphocytes thanks to the envelope glycoprotein gp120 [1,2]. If untreated, HIV can progress to AIDS (acquired immunodeficiency syndrome), which is characterized by a severe immune system dysfunction [3].

AIDS can predispose to the onset of various pathological conditions, including opportunistic infections and cancers [4,5,6]. Among the cancers, the one most commonly related to AIDS is Kaposi’s Sarcoma, caused by human herpesvirus 8 (HHV-8) [7]. Despite that, the onset of non-Hodgkin lymphomas (NHLs), especially particularly aggressive forms, such as the diffuse large B-cell lymphoma, is considered quite frequent [8]. It is known that DLBCL is one of the most common NLHs in adults, but disseminated extranodal disease is much less frequent, usually occurring in the advanced stages of the disease [9,10].

Based on the above, although AIDS-related DLBCL is considered a relatively frequent finding, images documenting both macroscopic and microscopic pluri-visceral extranodal involvement are extremely rare in the literature. In fact, despite the prolific clinical literature reporting the morphological features of biopsy and cytological sampling, there are very few postmortem reports that deal with this pathology, and the majority concern microscopic findings of the intravascular large B-cell lymphoma, a subtype of DLBCL [11,12].

Thus, the authors first propose the unique findings obtained during both the gross examination and the routine histology (hematoxylin-eosin staining).

In fact, in the case here presented, the autopsy revealed scattered lymph-adenomegalies and the presence of whitish nodular formations with increased consistency in the lungs, pericardium (Figure 1 and Figure 2), heart (Figure 3), liver (Figure 4), stomach (Figure 5) and kidneys (Figure 6).

Significant gross findings were observed in the lungs, especially in the right one, which showed a severe alteration of the morphology due to the confluence of the nodularities in large neoformations replacing/infiltrating most of the parenchyma (Figure 2). Moreover, large thrombi in the lumen of the pulmonary artery were observed.

Routine histology made it possible to define the nature of the whitish neoformations as extra-lymph node localizations of DLBCL. In particular, histological sections of the lungs (Figure 2), pericardium, heart (Figure 3), kidneys (Figure 5) and stomach (Figure 6) showed interstitial lymphoid proliferative infiltrates. The most involved organs were the lungs in which, as documented at the gross examination, multiple and diffuse/large neoplastic localizations were observed. Then, multiple pulmonary arteriolar thromboemboli were found (Figure 2).

The cause of death was an acute cardio-respiratory failure due to severe pulmonary thromboembolism, which occurred in a man affected by an “undiagnosed” DLBCL with extranodal localizations mainly determining the upheaval of the lungs’ architecture.

The contribution, mediated by DLBCL through the release of cytokines, procoagulant substances and the activation of coagulation factors by neoplastic cells, was significant in the cause of pulmonary thromboembolism.

## Figures and Tables

**Figure 1:**
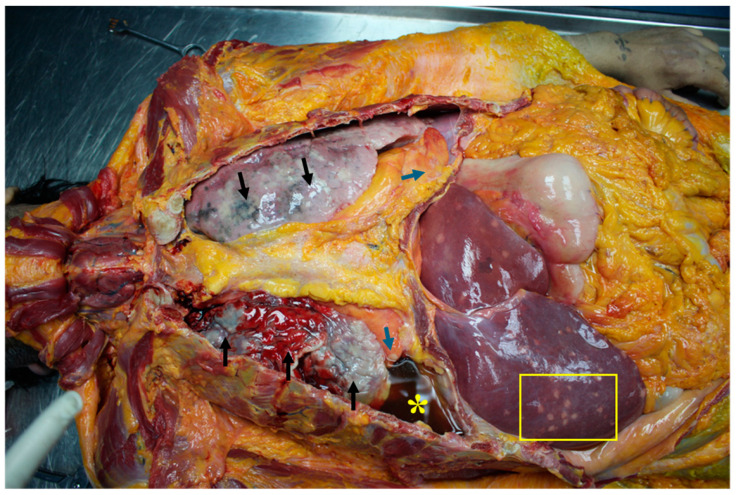
Thoracoabdominal region after the removal of skin, muscles and sternal plastron. It is possible to appreciate the presence of numerous and widespread whitish areas in the lungs (black arrows), pericardium (blue arrows) and liver (yellow square), due to the spread of DLBCL; the right lung appears small in size and floating in reddish pleural fluid (yellow asterisk).

**Figure 2:**
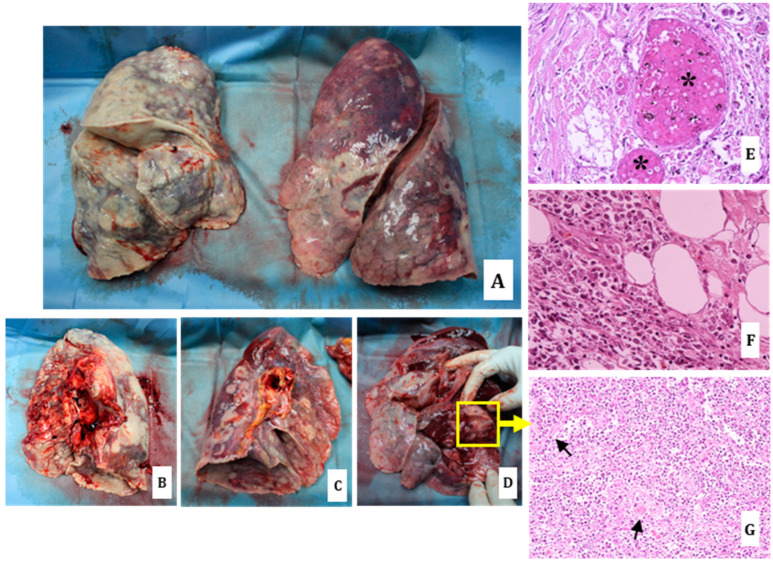
Lungs: significantly increased consistency of the parenchyma, showing irregular surface because of the presence of whitish nodules, diffusely affecting the entire right lung and predominantly the lower regions of the left lung (**A**–**C**). The same whitish areas were also documented at the cut surface in both lungs’ parenchyma ((**D**) left lung; yellow square). At routine histology, both multiple arteriolar thromboemboli ((**E**) hematoxylin-eosin staining, 40× black asterisks) and multiple localizations of the disease with interstitial lymphoid proliferative infiltrates predominantly involving the para-hilar serosa ((**F**) hematoxylin-eosin staining, 40×) and widely the congested lungs subserosa ((**G**) hematoxylin-eosin staining, 20×; black arrows for congested vessels).

**Figure 3:**
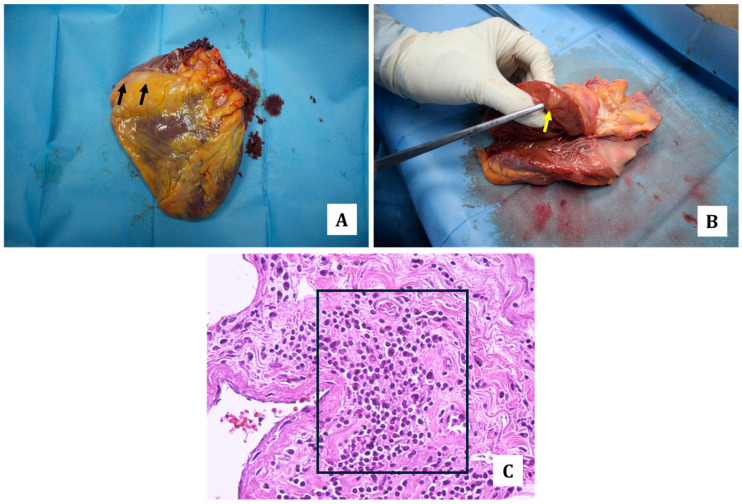
Heart: whitish and irregularly oval shape areas at the upper and anterior surface of right ventricle ((**A**) black arrows), and into the thickness of the left postero-lateral ventricle wall ((**B**) yellow arrow). At routine histology, multiple areas of DLBCL at the subepicardial regions with interstitial lymphoid proliferative infiltrates ((**C**) hematoxylin-eosin staining, 40×; black square;).

**Figure 4:**
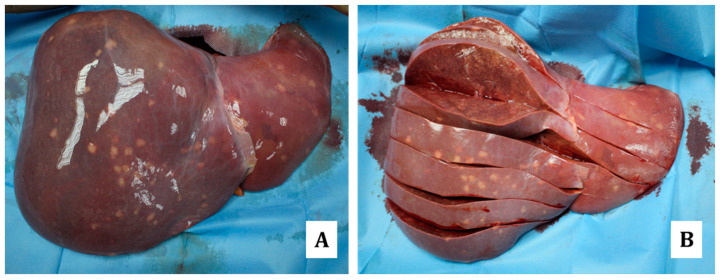
Liver: normal shape with scattered, whitish nodular areas, mostly sub-centimetric (deepening approximately 3 mm inside the parenchyma) and mainly localized on the anterior liver surface (**A**,**B**).

**Figure 5:**
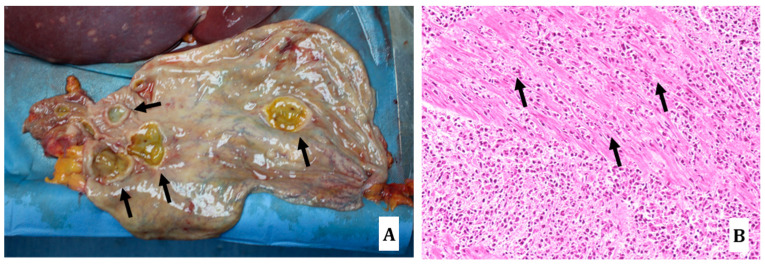
Stomach: neoplastic areas determining gastric irregularly thickened gastric wall with associated yellowish mucosal crater-shaped lesions of various sizes ((**A**) black arrows). At routine histology, widespread neoplastic localizations of DLBCL, with parietal muscular serous, subserous and extrinsic mural localizations characterized by widespread interstitial proliferative lymphoid infiltrates ((**B**) hematoxylin-eosin staining, 20×; black arrows to highlight the muscular layer;).

**Figure 6:**
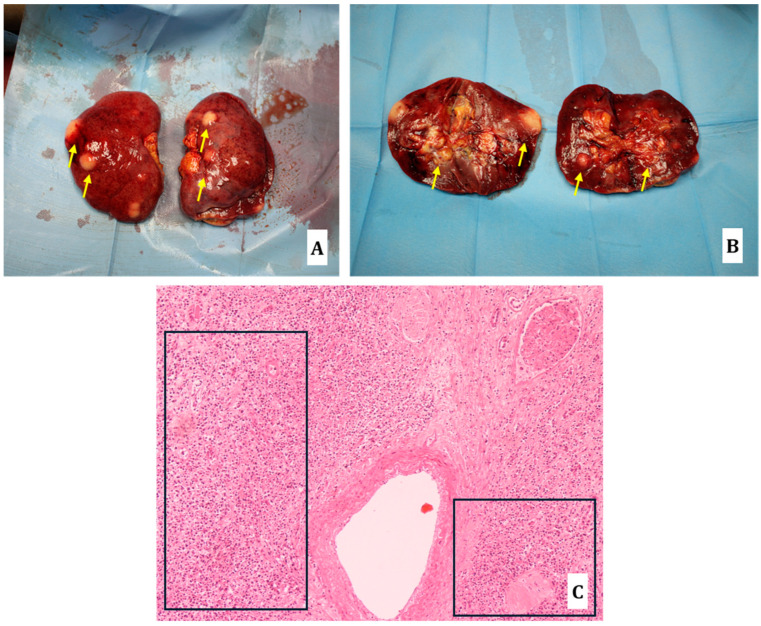
Kidneys: subcapsular whitish nodularity of increased consistency, with a maximum diameter of 20 mm ((**A**) yellow arrows); similar findings at the cutting surface ((**B**) yellow arrows). Routine histology showed multiple localizations of lymphomatous proliferative nodules ((**C**) hematoxylin-eosin staining, 10×; black squares).

## Data Availability

No new data were created or analyzed in this study.

## References

[1] Xu Y., Ollerton M.T., Connick E. (2019). Follicular T-cell subsets in HIV infection: Recent advances in pathogenesis research. Curr. Opin. HIV AIDS.

[2] Yoon V., Fridkis-Hareli M., Munisamy S., Lee J., Anastasiades D., Stevceva L. (2010). The GP120 molecule of HIV-1 and its interaction with T cells. Curr. Med. Chem..

[3] Yang X., Su B., Zhang X., Liu Y., Wu H., Zhang T. (2020). Incomplete immune reconstitution in HIV/AIDS patients on antiretroviral therapy: Challenges of immunological non-responders. J. Leukoc. Biol..

[4] Vangipuram R., Tyring S.K. (2019). AIDS-Associated Malignancies. Cancer Treat. Res..

[5] Yu L., Wan H., Shi J., Zhang B., Wang M. (2023). Disseminated Mycobacterium thermoresistibile Infection presented with Lymphadenectasis in an AIDS patient: Case report and review of literature. BMC Infect. Dis..

[6] Udoakang A.J., Djomkam Zune A.L., Tapela K., Nganyewo N.N., Olisaka F.N., Anyigba C.A., Tawiah-Eshun S., Owusu I.A., Paemka L., Awandare G.A. (2023). The COVID-19, tuberculosis and HIV/AIDS: Ménage à Trois. Front. Immunol..

[7] Muggia F.M., Lonberg M. (1986). Kaposi’s sarcoma and AIDS. Med. Clin. N. Am..

[8] Wang S.S. (2023). Epidemiology and etiology of diffuse large B-cell lymphoma. Semin. Hematol..

[9] Susanibar-Adaniya S., Barta S.K. (2021). 2021 Update on diffuse large B cell lymphoma: A review of current data and potential applications on risk stratification and management. Am. J. Hematol..

[10] Fiegl M., Greil R., Pechlaner C., Krugmann J., Dirnhofer S. (2002). Intravascular large B-cell lymphoma with a fulminant clinical course: A case report with definite diagnosis post mortem. Ann. Oncol..

[11] Asif A.A., Tharoor M., Fischer J.L. (2022). Intravascular Large B Cell Lymphoma—Still a Diagnostic Dilemma. J. Community Hosp. Intern. Med. Perspect..

[12] Maiese A., La Russa R., De Matteis A., Frati P., Fineschi V. (2020). Post-mortem diagnosis of intravascular large B-cell lymphoma. J. Int. Med. Res..

